# Posterior Approach Partial Mastectomy (MAPP): Early Clinical Experience with a Novel Oncoplastic Technique

**DOI:** 10.3390/jcm15082925

**Published:** 2026-04-12

**Authors:** Ahmad Kaviani, Gladys Bruyninx, Erica Patocskai

**Affiliations:** 1 Surgical Oncology Division, Department of Surgery, University of Montreal, Montreal, QC H3T 1J4, Canada; 2 University of Montreal Hospital Center (CHUM), 1000 Saint-Denis St., Montreal, QC H2X 0C1, Canada

**Keywords:** breast neoplasms, segmental mastectomy, oncoplastic surgery, minimally invasive surgical procedures

## Abstract

**Background:** Oncoplastic breast surgery aims to combine oncologic safety with optimal cosmetic outcomes. However, many established techniques require visible anterior breast incisions or substantial tissue rearrangement, which may compromise cosmetic results in selected patients. Posterior access to the breast through the retromammary space may allow tumor excision while preserving the anterior breast envelope. **Methods:** We report an early clinical experience with Posterior Approach Partial Mastectomy (MAPP), a breast-conserving technique that accesses the lesion through a concealed inframammary or lateral breast crease incision. This single-center retrospective case series included consecutive patients undergoing excision using this approach. Patient selection, surgical technique, and early outcomes—including margin status, complications, and need for re-excision—were evaluated. **Results:** Eight patients underwent breast-conserving excision using the MAPP technique. Six patients had malignant lesions (invasive ductal carcinoma with or without ductal carcinoma in situ or pure DCIS), while two benign lesions were included for technical completeness. Tumor size ranged from 9 to 78 mm. All malignant cases achieved negative surgical margins (R0), and no patient required re-excision. Posterior access was successfully achieved in all cases using concealed inframammary or lateral crease incisions. One patient experienced minor wound discharge that resolved with conservative management, and no major postoperative complications were observed. Follow-up ranged from 2 to 12 months. **Conclusions:** Posterior Approach Partial Mastectomy appears to be a feasible oncoplastic approach with encouraging early oncologic outcomes in carefully selected patients undergoing breast-conserving surgery. By preserving the anterior skin envelope and concealing the surgical incision, this technique may offer cosmetic advantages while maintaining oncologic adequacy. Larger studies with longer follow-up are needed to further define its role in oncoplastic breast surgery.

## 1. Introduction

Breast cancer remains the most common malignancy among women worldwide, with approximately one in seven women developing the disease during their lifetime [1]. Despite major advances in diagnostic modalities and systemic therapies, surgery continues to represent the cornerstone of curative treatment for early-stage disease [2]. Over recent decades, surgical strategies have progressively evolved from an exclusive focus on oncologic safety toward approaches that also prioritize quality of life and aesthetic outcomes [3,4]. Within this paradigm shift, cosmetic outcomes have emerged as a key determinant of patient satisfaction, psychological well-being, and long-term adherence to treatment [5,6].

Oncoplastic breast surgery (OBS) was developed to address these dual objectives by integrating oncologic resection with reconstructive principles. By allowing wider excisions while preserving breast appearance, OBS has been associated with improved margin status, reduced re-excision rates, and higher patient-reported satisfaction compared with conventional breast-conserving surgery [7,8]. However, many commonly used oncoplastic techniques—particularly volume-displacement and reduction-based approaches—still require visible anterior incisions, nipple–areola complex modifications, or significant alterations in breast volume and contour [9,10]. These changes may be especially undesirable for younger patients and for women with small to moderate breast volumes, in whom even limited tissue displacement can result in disproportionate cosmetic deformity and psychosocial impact [11,12,13].

In response to these limitations, alternative access strategies aimed at preserving the anterior breast envelope have gained interest. Posterior retromammary approaches offer the theoretical advantage of minimizing visible scarring while maintaining breast contour; however, clinical data supporting their feasibility and reproducibility in routine breast-conserving surgery remain limited. In particular, standardized descriptions of patient selection, technical execution, and early outcomes for posterior approaches are limited in the current literature.

Posterior Approach Partial Mastectomy (MAPP)was developed as a structured and reproducible application of the retromammary access approach within contemporary oncoplastic practice. This technique utilizes a concealed incision within the inframammary fold or lateral breast crease to access the lesion through the retromammary space. By preserving the anterior skin envelope and glandular architecture, this approach aims to minimize visible scarring and aesthetic compromise while adhering to established oncologic principles. In addition, this approach aligns with broader trends toward less invasive surgical strategies [14,15].

In this study, we report our early clinical feasibility experience with MAPP in a consecutive series of patients undergoing breast-conserving surgery for carefully selected breast lesions. We describe patient selection criteria, surgical technique, and early surgical and oncologic outcomes, with the objective of evaluating the feasibility and safety of this posterior oncoplastic approach in routine clinical practice.

## 2. Methods and Materials

### 2.1. Study Design and Setting

This single-center retrospective case series was conducted at the Surgical Oncology Division, Department of Surgery, University of Montreal (CHUM), to evaluate the technical feasibility and early clinical outcomes of a posterior retromammary approach for breast-conserving surgery termed Posterior Approach Partial Mastectomy (MAPP). Consecutive patients undergoing excision using this technique during the study period were included.

### 2.2. Patient Selection

Patients were considered eligible for MAPP if they had a breast lesion oncologically eligible for breast-conserving surgery (according to NCCN criteria) and for which a posterior retromammary approach was deemed technically feasible based on tumor location, depth, and accessibility. In general, appropriate candidates had small to moderate-sized lesions located in the inferior, lateral, or central breast regions and situated more than 1 cm from the skin surface, as determined by preoperative imaging.

Exclusion criteria included lesions requiring skin excision, tumors located < 1 cm from the skin surface, lesions in superior quadrants where posterior visualization would be limited, large tumors requiring wide resections (in case of malignant pathology), and patients with macromastia in whom reduction-based oncoplastic approaches would be more appropriate.

During the study period, two benign lesions were excised using the same posterior approach. These cases were included solely to illustrate technical feasibility and reproducibility and were not considered in the assessment of oncologic outcomes.

### 2.3. Preoperative Evaluation

All patients underwent clinical examination and standard imaging (mammography and/or ultrasonography; MRI when clinically indicated). Histologic diagnosis was established by core needle biopsy when appropriate. For non-palpable lesions, localization was performed according to institutional practice (wire localization, radioactive seed, Magseed^®^, or SAVI SCOUT^®^ system). The surgical approach was selected after tumor localization, and radiologic localization techniques were not modified for this study. Tumor–skin distance was measured preoperatively using mammography and/or ultrasonography.

### 2.4. Surgical Technique

#### 2.4.1. Conceptual Rationale

**Figure 1:**
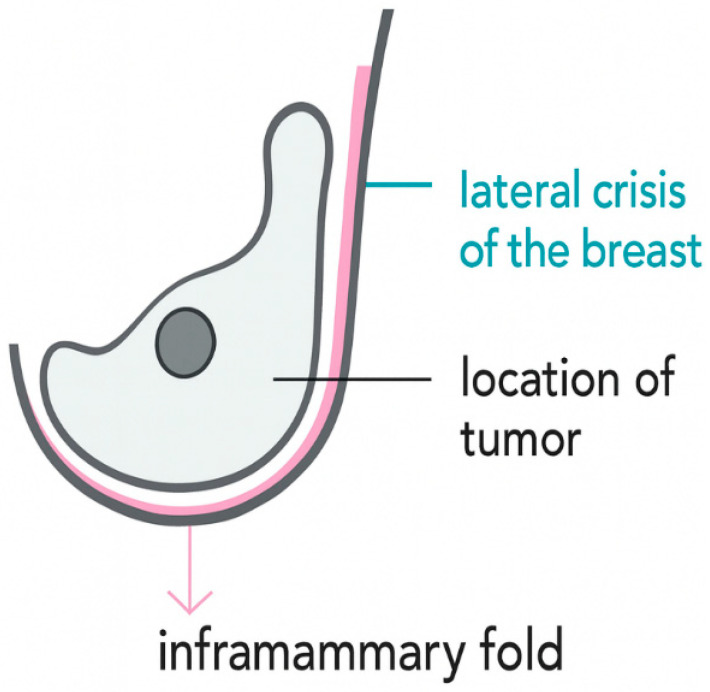
Schematic representation of tumor location in the breast.

The MAPP technique is based on accessing the lesion through the retromammary (pre-pectoral) space rather than via the anterior breast parenchyma. A schematic representation of tumor positioning relative to posterior access is shown in Figure 1.

#### 2.4.2. Operative Steps

**Figure 2:**
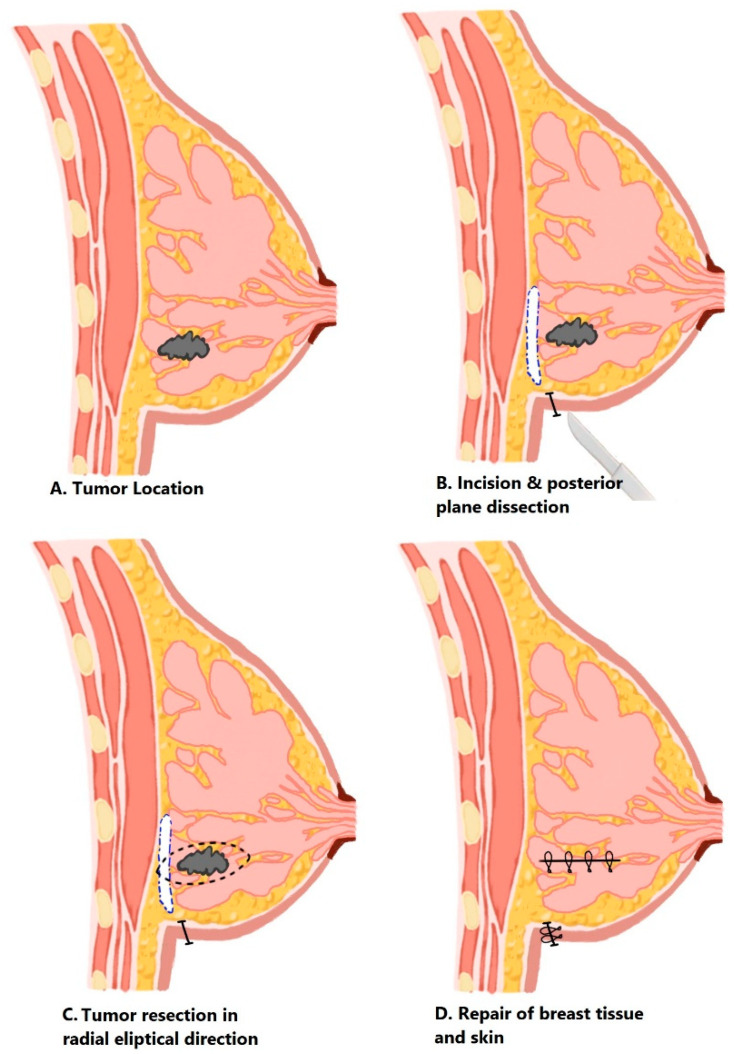
Stepwise illustration of the MAPP technique.

Procedures were performed under general anesthesia. Patients were positioned supine or in lateral decubitus depending on tumor location. A concealed incision (approximately 3–4 cm) was placed along the inframammary fold (IMF) or lateral/inferolateral breast crease, selected according to lesion position and cosmetic considerations (Figure 2A). Lesions located in the inferior breast were preferentially approached through the inframammary fold, whereas lateral or superolateral lesions were accessed through a lateral or inferolateral breast crease to optimize exposure and minimize visible scarring.

Dissection proceeded in a subfascial plane to enter the retromammary space (Figure 2B). The posterior surface of the breast gland was gently elevated from the pectoralis major fascia, allowing for a posterior approach to the lesion while preserving the anterior skin envelope and minimizing disruption of the superficial glandular architecture. In contrast to a narrow tunneling approach, dissection was performed in a fan-shaped manner within the retromammary space, providing broader exposure of the posterior breast surface and facilitating safe access to the lesion.

To facilitate adequate exposure through the limited posterior incision, retractors with longer blades were used to maintain visualization of the retromammary plane. Dissection and mobilization of the gland from the pectoralis fascia were performed primarily using electrocautery, allowing controlled hemostasis and precise tissue separation.

For lesions localized using radioactive seed localization, intraoperative identification of the seed was achieved using a gamma detection probe. Two complementary strategies were used to identify the seed location. In the first approach, the approximate position of the seed was identified externally using the gamma probe, and the surrounding tissue was gently stabilized between the surgeon’s fingers; the corresponding internal point within the retromammary space was then marked using a sterile surgical marker. Alternatively, angled gamma probes were introduced into the retromammary space to directly detect the radioactive signal and guide precise localization of the seed from within the posterior dissection plane.

Tumor excision was then performed with attention to achieving adequate oncologic margins (Figure 2C). All specimens were oriented after excision using sutures, and specimen mammography was routinely performed to confirm retrieval of the target lesion and to assess the adequacy of surgical margins. Intraoperative findings were used to guide margin evaluations when appropriate.

Following hemostasis, the resection cavity was closed with radial interrupted sutures to minimize dead space and reduce contour deformity. Skin closure was achieved using absorbable intradermal sutures or adhesive strips, preserving the concealed scar within the natural fold (Figure 2D). Drains were not routinely placed.

### 2.5. Outcomes

The primary objective of this study was technical feasibility, defined as successful completion of posterior access excision with satisfactory wound closure.

Early clinical outcomes included:

- Pathologic margin status, reported from final pathology (R0 defined according to institutional standards).
- Need for re-excision, defined as return to the operating room for additional margin excision following the index procedure.
- Postoperative complications, recorded during follow-up visits.
- Follow-up duration, calculated from surgery to the most recent clinical evaluation.

Clinicopathologic characteristics and early surgical outcomes are summarized in Table 1.

Given the exploratory nature and limited sample size, the analyses were descriptive.

**Figure 3:**
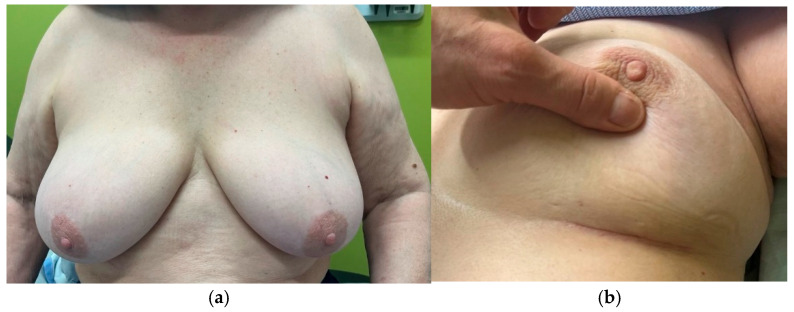
Clinical outcome of the MAPP technique in a 71-year-old patient, 4 months post-surgery and ultra-hypofractionated radiotherapy. (**a**): Sitting position. (**b**): Supine position showing concealed incision.

A representative postoperative cosmetic outcome is shown in Figure 3**,** illustrating a representative postoperative appearance with a concealed incision at follow-up.

### 2.6. Ethics

All procedures were performed in accordance with institutional standards and the Declaration of Helsinki. Written informed consent was obtained for surgery and for use of anonymized clinical information and images when applicable.

## 3. Results

A total of eight patients underwent breast-conserving excision using the Posterior Approach Partial Mastectomy (MAPP) technique during the study period. Clinicopathologic characteristics and early surgical outcomes are summarized in Table 1.

Patient age ranged from 27 to 83 years. Six patients had malignant lesions, including invasive ductal carcinoma (IDC) with or without ductal carcinoma in situ (DCIS), or pure DCIS. Two patients had benign lesions (lipoma and fibroadenoma), which were included for technical completeness. Tumor size ranged from 9 mm to 78 mm. Lesions were most commonly located in the lower outer quadrant (LOQ) and lower inner quadrant (LIQ), with additional cases in the upper outer quadrant and central breast region (Table 1). Preoperative tumor–skin distance, as assessed by imaging, ranged from 18 mm to 38 mm.

All malignant cases achieved negative surgical margins (R0 resection) on final pathology (Table 1). Margin widths ranged from 2 mm to 6 mm, with one case demonstrating a 1 mm margin supplemented by cavity shave excision. No patient required re-excision following the initial procedure (0/8).

Posterior access was successfully achieved in all cases through a concealed incision placed along the inframammary fold, lateral crease, or inferolateral crease, depending on tumor location (Table 1). Drains were not routinely used.

Postoperative recovery was uneventful in the majority of patients. One patient developed minor wound discharge, which resolved with conservative management. No major postoperative complications were observed during the follow-up period. The follow-up duration ranged from 2 to 12 months (Table 1).

## 4. Discussion

Oncoplastic breast surgery has reshaped the surgical management of breast cancer by combining oncologic safety with improved cosmetic and psychosocial outcomes, leading to higher patient satisfaction and quality of life [6,8,16]. Nevertheless, commonly used oncoplastic techniques are not without limitations. Conventional breast-conserving surgery and Level I oncoplastic approaches often result in visible anterior breast scars, which may negatively influence body image and long-term patient perception of surgical outcomes [10,16]. More advanced Level II techniques, including therapeutic reduction mammoplasty, may significantly alter breast volume and symmetry and frequently require contralateral symmetrization. These procedures are less suitable for women with small to moderate breast volumes, in whom limited tissue displacement increases the risk of noticeable deformity [11,12,13,17].

Although retromammary access to the breast has occasionally been used in selected cases, particularly for benign lesions, its application and indications have not been standardized or systematically described in the literature. Posterior Approach Partial Mastectomy (MAPP) was developed to address these limitations by accessing breast lesions through the retromammary space via a concealed incision placed within the inframammary fold or lateral breast crease. In this early clinical experience, posterior access was successfully achieved in all cases, with preservation of the anterior skin envelope and breast contour. The incision was consistently concealed within natural breast folds, resulting in the absence of visible anterior breast scars during the follow-up period. Concealed-scar techniques have been associated with improved patient satisfaction and cosmetic perception in breast surgery [16,17]. However, cosmetic outcomes in the present study are descriptive and were not assessed using objective or validated measures.

From an oncologic perspective, all malignant lesions in this series were excised with negative surgical margins, and no patient required re-excision. These findings suggest that oncologic adequacy may be achievable using a posterior approach in carefully selected patients. Margin status and re-excision rates observed in this series are consistent with those reported for standard breast-conserving and oncoplastic procedures in the literature [7,8,18]. In addition, the observed complication profile was limited to minor events, with no major postoperative complications. This aligns with published data indicating that oncoplastic techniques, when appropriately selected, do not increase surgical morbidity compared with conventional breast-conserving surgery [10,18].

This study represents a small early feasibility case series, and its primary objective was to evaluate the technical applicability and safety of the MAPP technique rather than to demonstrate oncologic or cosmetic superiority. The inclusion of two benign lesions reflects the feasibility-oriented nature of this study. These lesions were excised using the same posterior approach and were included solely to demonstrate the technical applicability and reproducibility of the approach across different breast pathologies; they were not considered in the interpretation of oncologic outcomes. Such inclusion is consistent with early surgical feasibility studies, in which technical execution and procedural safety are prioritized over comparative outcome analysis.

The posterior access strategy employed in MAPP may be particularly advantageous for lesions located in the inferior, lateral, and central breast regions, where anterior lumpectomy incisions are more likely to result in contour deformities. By preserving anterior glandular architecture and avoiding nipple–areola complex manipulation or contralateral symmetrization, MAPP occupies an intermediate position between simple lumpectomy and reduction-based oncoplastic procedures. This approach may therefore represent a potential alternative for selected patients with small to moderate breast volumes who prioritize minimal scarring and preservation of natural breast shape.

Several limitations should be acknowledged. The small sample size, limited follow-up duration, absence of validated patient-reported outcome measures, particularly for aesthetic evaluation, and lack of a comparative control group restrict the generalizability of these findings. Consequently, conclusions regarding long-term oncologic outcomes, durability of cosmetic results, and comparative effectiveness cannot be drawn from this early experience. Given these limitations, the present study should be interpreted as an early technical feasibility series rather than evidence of oncologic or cosmetic superiority.

Beyond its immediate clinical application, the posterior retromammary access utilized in MAPP may provide a conceptual framework for future minimally invasive approaches or endoscopic-assisted breast surgery. Prior studies have highlighted growing interest in minimally invasive breast surgical approaches; however, such techniques remain technically demanding and are not yet widely adopted [14,15]. Any adaptation of MAPP to endoscopic, laparoscopic, or robotic platforms remains speculative and would require dedicated technical development and prospective validation.

In summary, this early case series suggests that Posterior Approach Partial Mastectomy is a technically feasible and reproducible surgical approach in carefully selected patients undergoing breast-conserving surgery. While early surgical outcomes are encouraging, further studies with larger cohorts, longer follow-up, and standardized outcome assessment are required to better define its role in clinical practice.

## 5. Conclusions

Posterior Approach Partial Mastectomy (MAPP) represents a feasible and reproducible surgical approach in selected patients undergoing breast-conserving surgery. This technique may offer potential cosmetic advantages while preserving oncologic principles. Larger studies with longer follow-up periods and objective outcome assessments are required to further validate this approach.

## Figures and Tables

**Table 1:** Clinicopathologic characteristics and early surgical outcomes of patients undergoing Posterior Approach Partial Mastectomy (MAPP), including benign cases for technical demonstration *.

	Age	Pathology	Tumor Location *	Tumor Size (mm)	Incision	Distance from Skin (mm) **	Margin Status	Re-Excision	Complication	Follow Up (Months)
1	27	lipoma	UOQ	78	Lateral crease	20	N/A	No	No	9
2	71	IDC + DCIS	LOQ	9	IMF ^‡^	38	R0 (2 mm)	No	No	15
3	75	IDC + DCIS	Lower Central	13	IMF	34	R0 (2 mm)	No	No	11
4	73	IDC + DCIS	LIQ	17	IMF	26	R0 (2 mm)	No	No	10
5	59	DCIS	LIQ	16	IMF	33	R0 (1 mm + cavity shave)	No	No	6
6	83	IDC	LOQ	8	IMF	30	R0 (6 mm)	No	No	4
7	61	DCIS	LOQ	14	Inferolateral crease	21	R0 (>4 mm)	No	Minor wound discharge	3
8	37	Hamartoma	True Lateral	37	Inferolateral crease	18	N/A	No	No	5

* Two cases were benign lesions excised using the same posterior approach and are included for technical demonstration only and not considered in oncologic outcome interpretation. ** Quadrants: LOQ (lower Outer Quadrant, LIQ (Lower Inner Quadrant), UOQ (Upper Outer Quadrant). ^‡^ Infra-Mammary Fold.

## Data Availability

The data presented in this study are available on reasonable request from the corresponding author. The data are not publicly available due to patient privacy considerations.
